# Bone mineral density in elite masters athletes: the effect of body composition and long-term exercise

**DOI:** 10.1186/s11556-021-00262-0

**Published:** 2021-05-31

**Authors:** Anna Kopiczko, Jakub Grzegorz Adamczyk, Karol Gryko, Marek Popowczak

**Affiliations:** 1 Department of Human Biology, Józef Piłsudski University of Physical Education in Warsaw, Marymoncka 34, 00-968 Warsaw, Poland; 2 Department of Theory of Sport, Józef Piłsudski University of Physical Education in Warsaw, Marymoncka 34, 00-968 Warsaw, Poland; 3 Department of Sport Games, Józef Piłsudski University of Physical Education in Warsaw, Marymoncka 34, 00-968 Warsaw, Poland; 4 Department of Team Sport Games, University School of Physical Education in Wrocław, Al. Ignacego Jana Paderewskiego 35, 51-612 Wrocław, Poland

**Keywords:** Bone mineral density, Body fat and lean body mass, Master athletes, Physical activity

## Abstract

**Background:**

The purpose of the study was to examine how bone mineral density (BMD) is related to body composition depending on the practiced sport (endurance, speed-power, throwing sports) in participants of the World Masters Athletics Championship.

**Methods:**

Dual-energy X-ray absorptiometry (DXA) was used to determine BMD and bone mass (BMC). Body composition was analyzed by means of the JAWON Medical X-scan analyzer using bioelectrical impedance methods. Percentage body fat (%BF), body fat mass (BFM), lean body mass (LBM), total body water (TBW), soft lean mass (SLM), intracellular water (ICW), and extracellular water (ECW) were evaluated.

**Results:**

Among men, the most important variables affecting the BMD norm were LBM (OR = 32.578; *p* = 0.023), ECW (OR = 0.003; *p* = 0.016) and ICW (OR = 0.011; *p* = 0.031), in the distal part and SLM (OR = 5.008; *p* = 0.020) and ICW (0.354, *p* = 0.008) in the proximal part. In women, the most important predictors of normal BMD were ICW (OR = 10.174; *p* = 0.003) and LBM (OR = 0.470; p = 0.020) in the distal part and ICW (OR = 5.254; *p* = 0.038) in the proximal part.

**Conclusion:**

The representatives of strength based events had the most advantageous BMD levels. The condition of bone tissue evaluated by BMC and BMD of the forearm in masters athletes was strongly determined by the level of lean body components and the type of sports training associated with the track and field event. In the most important predictors of the BMD norm were also hydration components ECW and ICW. However, this relationship requires more research on the nature and mechanisms of these interactions.

## Introduction

Aging is accompanied by adverse changes in body functioning, including those associated with impaired bone metabolism. Across the lifespan, bone adapts both shape and structure to changing loads and body functions [1]. However, adaptability decreases with aging. Prospective studies have shown that the risk of fracture increases progressively with increased age and decreased bone mineral density (BMD) [1].

From 25 years of age, humans lose about 1% of their bone mass per year [2]. The main causes of the age-related decline in bone mineral density are reduced supply and absorption of calcium, hormonal changes, and decreasing levels of physical activity [2]. However, these causes may differ between males and females with decreased BMD observed to greater extent in postmenopausal women. In women, estrogen is very important for maintaining or increasing bone density. Furthermore, its level is associated with reduced skeletal blood flow, physical inactivity, insufficient calcium intake, and decreased absorption by the gut, a reduced hormone function, and genetics [3]. Besides reduced levels of physical activity, several factors are believed to cause age-related loss in bone density in men. These include decreased levels of gender hormones and insulin-like growth factor (IGF-1) and nutrition lacking minerals. Furthermore, secondary osteoporosis may be due to several acquired habits and inherited conditions [2].

Regardless of gender, physical activity seems to be an important factor affecting bone health. However, to date there is no consensus as to the type and volume of physical activity required to normal BMD into older age. Both weight-bearing exercise that forces the body to work against gravity, and weight training have been shown to directly increase bone mineral density due to higher forces acting on muscles, tendons, ligaments, and bone, causing the bones to remodel and get stronger [3].

Thus, exercise programmes for older individuals should include strength, aerobic, and high-impact weight-bearing training which may help improve or counteract the decrease in bone mineral density observed with aging [4]. Indeed, Nikander et al. [5] suggested that exercise regimens that include stimulus between moderate to high impacts from different loading directions may be the optimal mode to improve bone structure and strength across the lifespan.

While weighted exercises can help maintain BMD in postmenopausal women and increase BMD of the spine and hip in women with osteopenia and osteoporosis [6], regular walking has no significant effect on the preservation of BMD at the spine, possibly due to insufficient stimuli [7, 8]. Nevertheless, regular resistance training and impact-loading activities should be considered the main strategy to prevent osteoporosis in older adults [9]. Mixed loading exercise programmes include combining jogging or other continuous forms of activity with other low-impact loading exercises and programmes that combine impact activity with high-magnitude exercise as resistance training appears to be effective in reducing postmenopausal bone loss at the hip and spine [7]. High-intensity training appears to be underestimated in reducing the risk of osteoporosis. Varahra et al. [10] recently suggested that a multicomponent exercise program of high-speed training combined with simulated functional tasks may enhance functional outcomes for people with osteoporosis. Moreover, previous research suggest that combining strength and conditioning exercise protocols (endurance and resistance training along with intensive work) is the best choice to preserve/improve bone mineral density in pre- and postmenopausal women as well as pre- and postandropausal men [11, 12].

As physical activity is strongly recommended for preventing osteoporosis, it can be assumed that people who are actively aging are also less susceptible to both osteopenia and osteoporosis. Thus, masters athletes should have greater bone mineral density than aged-matched non-athletes and inactive peers [13, 14]. Masters athletes are usually defined as those older than 35 years, as this is the age at which cardiovascular issues tend to show greater morbidity [15]. It might be suggested that aging athletes who are less aerobically fit have lower bone density, are at higher risk of some degenerative joint diseases, and suffer more injuries. Loss of bone mineral density is not completely reversible so older female masters athletes approaching menopause need to be more careful. Rapid mass loss in males can also cause several disorders including a drop of muscle mass, abilities to recover after training, and performance itself [3].

Therefore, the main purpose of the study was to exam how bone mineral density is related to body composition depending on the sport’s type (endurance, speed-power, throwing sports) in participants of the World Masters Athletics Championship. As a secondary objective, we aimed to determine which factors are most important for achieving normal BMD values.

## Methodology

### Sample and procedure

The study included 244 participants at the World Masters Athletics Championship held in March 2019 in Poland (107 women aged 56.6 ± 11.3 years and 137 men aged 57.0 ± 11.9 years). Inclusion criteria were as follows: written consent to participate in the study and the lack of health contraindications to densitometry and body composition analysis. Women using hormonal therapy were excluded from the study.

The athletes were divided into 3 categories according to the declared type of event during the competition: endurance athletes (EA: long-distance running > 400 m, steeplechase running, marathon), speed-power athletes (SPA: sprint ≤400 m, high jump, long jump, hurdles, pentathlon, triple jump), and throwing athletes (TA: hammer throw, discus, shot put, javelin throw, weight throw).

The study analyzed medallists of both European and world championships. All were regular participants of international athletic championships had trained in athletics since their youth and reported a current training frequency of at least four times a week.

### Dual-energy X-ray absorptiometry

Bone parameters of the non-dominant forearm were measured by the densitometric method for measuring the peripheral skeleton. The method used was dual-energy X-ray absorptiometry. Bone parameters such as bone mass (BMC in grams) and bone mineral density (BMD in gram/cm^2^) were measured. The measurements were taken at two locations on the forearm (distal and proximal) and the results were given for radiale plus ulna (R + U) and only radiale (R). The proximal site spans 10 mm starting at the 1/3 forearm length and continuing proximally. A Norland (Swissray-USA, Norland Medical Systems Madison WI, USA) densitometer was used, with the effective dose (μSv) of 0.05. We reported results as T-scores (the indicator that compares bone density of the individual with the mean of the population of healthy young people). The densitometer was calibrated by means of original phantoms recommended by the manufacturer [16].

### Bioelectrical impedance methods

Body composition was analyzed by means of the JAWON Medical X-scan PLUS 970 (Jawon Medical Co.,Ltd., Seul, South Korea) analyzer using bioelectrical impedance methods. Body mass, percentage body fat (%BF), body fat mass (BFM), lean body mass (LBM), total body water (TBW), soft lean mass (SLM), intracellular water (ICW), and extracellular water (ECW) were evaluated. The BIA analyzer was calibrated each morning prior to each analysis. Basic body dimensions and indices were evaluated with the use of anthropometric measurements. The length of the forearm was measured using large anthropometric calipers at the radiale-stylion points (r-sty) (1 mm, GPM Spreading Calliper, previous brand name Siber Hegner, Switzerland). Body height was measured without shoes with an anthropometer with a measurement accuracy of 1 mm (GPM Anthropometer, Switzerland). The data on health, training, and physical activity history were obtained by means of a short structured interview, administered by one of the researchers to each participant.

### Statistical analysis

The normality of distribution was verified by the Shapiro-Wilk test and the assumption of equality of variances assessed with the Levene test of homogeneity of variance. The data analysis was based on the factor analysis of variance (ANOVA) and the Bonferroni (post hoc) test. Effect size was calculated as eta-squared (η^2^) (small effect < 0.06; medium effect 0.06–0.14; large effect > 0.14) [17, 18]. The chi-squared test (χ^2^) was used to assess the differences in the frequency of occurrence of normal and low BMD in men and women according to the event type. To determine the effect size for the chi-squared test, the phi factor (Φ) was used (small effect: 0.1; medium effect: 0.3; large effect: 0.5) [19]. Furthermore, multivariate analysis (backward stepwise logistic regression; input *p* = 0.001 and removing *p* = 0.150) was used to indicate an independent association of the correct bone tissue mineralization with individual factors. The likelihood of normal bone mineralization (odds ratio - OR) was evaluated with a 95% confidence interval. The same number of predictors was included in all models. The tables with the regression results consider the predictors that have entered the model. The logistic regression model used the R^2^ Nagelkerke coefficient formula. The significance of the regression coefficient was assessed using the Wald-Wolfowitz series test. In all the analyses, the significance of the effects was assumed at *p* < 0.05. All calculations were performed with STATISTICA software (v. 12.0, StatSoft, USA).

## Results

Table 1 shows the prevalence of normal and underestimated bone mineralization in individual parts of the forearm according to gender and sport. In women from the TA group, significantly more frequent (χ2 = 19.7; *p* < 0.001; Φ = 0.4; medium effect) normal bone density was found in the proximal part of radial plus ulna (R + U) compared to EA and SPA (by 50.7 and 23.2%, respectively). In the same group, a higher percentage of normal bone density (ranging from 32 to 40%) was recorded in the distal part of radial plus ulna (χ2 = 9.8; *p* = 0.043; Φ = 0.3; medium effect). It was also observed that 87.5% of women in the EA group and 60% in the SPA group were characterized by underestimated BMD values (osteopenia + osteoporosis) in the proximal part of radial plus ulna (R + U).

**Table 1:** The frequency of occurrence of normal and below-normal bone mineral status in men and women (%) depending on the sport and percentage differences (χ^2^ test, level of significance p)

Bone Mineral Density	Reference ranges	Men (***n*** = 137)			χ^**2**^ (***p***) Φ	Women (***n*** = 107)			χ^**2**^ (***p***) Φ
		EA	SPA	TA		EA	SPA	TA	
Distal R + U	Normal (T-score = > −0.99)	69.4	77.8	100	9.2 (0.057) 0.3	50.0	57.5	89.5	9.9 (0.043) 0.3
	Osteopenic (T-score − 1 to −2.49)	29.0	22.2	–		47.9	42.5	10.5	
	Osteoporosis (T-score < = − 2.50)	1.6	–	–		2.1	–	–	
Proximal R + U	Normal (T-score= > −0.99)	46.8	44.4	61.9	3.8 (0.431) 0.2	12.5	40.0	63.2	19.7 (< 0.001) 0.4
	Osteopenic (T-score − 1 to −2.49)	40.3	44.4	38.1		37.5	22.5	26.3	
	Osteoporosis (T-score < = − 2.50)	12.9	11.2	–		50.0	37.5	10.5	
Proximal R	Normal (T-score = > −0.99)	62.9	68.5	85.7	4.1 (0.392) 0.2	33.3	47.5	68.4	9.0 (0.060) 0.3
	Osteopenic (T-score − 1 to −2.49)	30.6	25.9	14.3		22.9	25.0	21.1	
	Osteoporosis (T-score < = − 2.50)	6.5	5.6	–		43.8	27.5	10.5	

Notes: *EA* Endurance athletes, *SPA* Speed-power athletes, *TA* Throwing athletes

In male master athletes, the prevalence of normal and below normal bone mineral status differed from the female group. There was a lower than women prevalence of osteopenia and osteoporosis regardless of athletic event. Osteoporosis had a low prevalence among EA and SPA. There were no cases of osteoporosis in the TA group. It was also observed that 30.6% of men in the EA group and 22.2% in the SPA group were characterized by underestimated BMD values (osteopenia + osteoporosis) in the distal part of radial plus ulna (R + U). In the proximal part, underestimated BMD was noted in a higher percentage of men, in the EA and SPA groups in more than half of the subjects.

The analysis of individual variables in male masters athletics is presented in Table 2. Considering the diversity of values describing men, a higher BMC was observed in the TA group compared to EA and SPA in the proximal part (R) (by 14.3 and 14.2%, respectively), R + U (by 12.4 and 10.8%, respectively), BMC in the distal part (by 18.3 and 14.6%, respectively), T-score in the distal part (by 0.934 and 0.641), % age matched in the distal part (by 14.8 and 10.1% respectively). Furthermore, significantly higher (28–59%) values for individual body composition components such as ECW, TBW, LBM, SLM, ICW, BFM were found in TA compared to EA and SPA. Also compared to EA and SPA, TA athletes were characterized by higher values of individual anthropometric characteristic, such as body height (by 4.1 and 3.4%, respectively), body mass (by 40.7 and 31.7%, respectively), wrist width (by 17.7 and 10.6%), forearm length (by 4.5 and 3.6%). On the other hand, in the SPA group compared to EA, 6.2% higher values of BMD were observed in the distal part of individual body components: LBM, TBW, SLM, ICW, ECW (6.6–7.1%) and anthropometric characteristics such as body mass, wrist width (by 6.8 and 6.4%, respectively).

**Table 2:** General characteristics of the analyzed variables in male Masters Athletes (*n* = 137)

	Endurance athletes (***n*** = 62)	Speed-power athletes (***n*** = 54)	Throwing athletes (***n*** = 21)	Significant difference	***F (p)***	***η***^***2***^
	Mean ± SD					
Age [years]	56.1 ± 11.8	57.9 ± 12.2	57.5 ± 12.0	–	0.4 (0.690)	0.01
Body mass [kg]	70.5 ± 8.0	75.3 ± 8.3	99.2 ± 15.9	1v2v3	69.5 (< 0.001)	0.51
Height [cm]	175.7 ± 5.6	177.0 ± 8.1	183.0 ± 7.3	1,2v3	8.8 (< 0.001)	0.12
Forearm length [cm]	26.4 ± 1.3	26.6 ± 1.5	27.6 ± 1.8	1,2v3	5.6 (0.005)	0.08
Wrist width [cm]	6.2 ± 0.98	6.6 ± 0.72	7.3 ± 0.89	1v2v3	12.3 (< 0.001)	0.15
BMD dis R + U [g/cm^2^]	0.420 ± 0.067	0.446 ± 0.076	0.488 ± 0.056	1v2	7.7 (< 0.001)	0.10
BMD prox R + U [g/cm^2^]	0.886 ± 0.087	0.888 ± 0.089	0.916 ± 0.047	–	1.1 (0.329)	0.02
BMD prox R [g/cm2]	0.902 ± 0.088	0.910 ± 0.093	0.928 ± 0.059	–	0.7 (0.487)	0.01
BMC dis R + U [g]	2.019 ± 0.357	2.085 ± 0.404	2.389 ± 0.256	1,2v3	8.2 (< 0.001)	0.11
BMC prox R + U [g]	2.631 ± 0.306	2.670 ± 0.343	2.958 ± 0.268	1,2v3	8.7 (< 0.001)	0.11
BMC prox R [g]	1.382 ± 0.151	1.384 ± 0.253	1.580 ± 0.162	1,2v3	8.7 (< 0.001)	0.11
T-score dis R + U	−0.442 ± 0.992	− 0.129 ± 1.044	0.512 ± 0.818	1,2v3	7.4 (0.001)	0.10
T-score prox R + U	−1.189 ± 0.978	−1.130 ± 0.967	−0.778 ± 0.529	–	1.6 (0.205)	0.02
T-score prox R	−0.766 ± 1.014	−0.690 ± 1.045	−0.459 ± 0.673	–	0.8 (0.467)	0.01
% age matched dis R + U	93.2 ± 15.2	97.9 ± 16.0	108.0 ± 12.7	1,2v3	7.5 (0.001)	0.10
% age matched prox R + U	89.4 ± 8.8	89.9 ± 8.5	92.5 ± 4.7	–	1.1 (0.326)	0.02
% age matched prox R	93.1 ± 9.1	94.0 ± 9.3	95.7 ± 5.8	–	0.7 (0.498)	0.01
%BF	16.6 ± 3.9	16.7 ± 5.5	18.1 ± 4.2	–	0.9 (0.425)	0.01
BFM [kg]	11.7 ± 3.4	12.7 ± 4.4	18.5 ± 6.6	1,2v3	18.8 (< 0.001)	0.22
LBM [kg]	59.1 ± 6.4	63.1 ± 8.7	81.8 ± 11.8	1v2v3	59.3 (< 0.001)	0.47
TBW [kg]	42.5 ± 4.6	45.5 ± 6.3	58.9 ± 8.5	1v2v3	59.4 (< 0.001)	0.47
SLM [kg]	54.9 ± 6.0	58.7 ± 8.2	76.0 ± 10.9	1v2v3	58.1 (< 0.001)	0.46
ICW	25.8 ± 3.0	27.6 ± 3.9	35.5 ± 5.3	1v2v3	52.2 (< 0.001)	0.44
ECW	16.7 ± 1.8	17.8 ± 2.4	23.4 ± 3.3	1v2v3	67.1 (< 0.001)	0.50

Notes: significant differences between groups (1 – endurance athletes; 2 – speed-power athletes, 3 – throwing athletes)

*BMD* Bone mineral density, *BMC* Bone mass, dis- distal part of forearm, *prox* proximal part of forearm, *BF* Body fat, *BFM* Body fat mass, *LBM* Lean body mass, *TBW* Total body water, *SLM* Soft lean mass, *ICW* Intercellular water, *ECW* Extracellular water

The analysis of individual variables in women is presented in Table 3. With regard to the most important variables in the TA group, higher values of BMD and BMC in the proximal part R + U (by 20.6 and 27.6%, respectively), BMD (by 18.5%) and BMC (by 28.4%) in the proximal part R. Identical trend was observed between TA and SPA in BMC R + U and R in the proximal part (17–18%). In terms of the differentiation of variables describing body composition components in TA group as compared to EA and SPA, higher TBW (by 84.9 and 70.9% respectively), LBM (by 33 and 23.1%, respectively), ICW (by 31.9 and 22%, respectively), ECW (by 50.8 and 22.9%, respectively), SLM (by 32.2 and 23.4%, respectively), BFM (by 78.5 and 66.9%, respectively), and %BF (by 6.1 and 5.9%, respectively) were recorded. Furthermore, compared to EA, athletes from the SPA group were characterized by higher values (7–10%) of LBM, TBW, ICW, ECW and SLM. The analysis of basic anthropometric characteristics in women who trained throwing sports compared to EA and SPA found greater body mass (by 47 and 38.3%, respectively) and wrist width (by 12.8 and 8.2%).

**Table 3:** General characteristics of the analyzed variables in female Masters Athletes (*n* = 107)

	Endurance athletes (***n*** = 48)	Speed-power athletes (***n*** = 40)	Throws athletes (***n*** = 19)	Significant difference	***F (p)***	***η***^***2***^
	Mean ± SD					
Age [years]	57.7 ± 11.1	55.2 ± 12.2	56.8 ± 9.8	–	0.6 (0.562)	0.01
Body mass [kg]	57.0 ± 7.3	60.6 ± 5.7	83.8 ± 12.2	1,2v3	82.5 (< 0.001)	0.61
Height [cm]	162.6 ± 6.8	166.2 ± 7.7	169.9 ± 5.4	1v3	8.1 (< 0.001)	0.13
Forearm length [cm]	24.4 ± 1.1	24.9 ± 1.3	25.1 ± 1.2	–	3.2 (0.046)	0.06
Wrist width [cm]	4.7 ± 0.3	4.9 ± 0.3	5.3 ± 0.6	1v2v3	20.8 (< 0.001)	0.28
BMD dis R + U [g/cm^2^]	0.293 ± 0.054	0.322 ± 0.075	0.350 ± 0.053	1v3	6.1 (0.003)	0.10
BMD prox R + U [g/cm^2^]	0.649 ± 0.101	0.713 ± 0.118	0.783 ± 0.094	1v2,3	11.5 (< 0.001)	0.18
BMD prox R [g/cm2]	0.677 ± 0.117	0.736 ± 0.118	0.802 ± 0.101	1v3	8.6 (< 0.001)	0.14
BMC dis R + U [g]	1.222 ± 0.265	1.323 ± 0.316	1.544 ± 0.245	1,2v3	8.9 (< 0.001)	0.15
BMC prox R + U [g]	1.718 ± 0.259	1.867 ± 0.298	2.192 ± 0.381	1,2v3	17.2 (< 0.001)	0.25
BMC prox R [g]	0.908 ± 0.140	0.995 ± 0.159	1.166 ± 0.202	1v2v3	17.8 (< 0.001)	0.25
T-score dis R + U	−0.902 ± 0.834	−0.547 ± 1.179	−0.031 ± 0.835	1v3	5.6 (0.005)	0.10
T-score prox R + U	−2.660 ± 1.605	−1.691 ± 1.859	−0.555 ± 1.494	1v2,3	11.2 (< 0.001)	0.18
T-score prox R	− 2.204 ± 1.840	−1.308 ± 1.863	− 0.223 ± 1.608	1v3	8.6 (< 0.001)	0.14
% age matched dis R + U	95.1 ± 15.3	100.6 ± 21.9	111.4 ± 17.8	1v3	5.3 (0.006)	0.09
% age matched prox R + U	87.7 ± 10.3	93.6 ± 13.7	103.8 ± 12.0	1,2v3	12.4 (< 0.001)	0.19
% age matched prox R	92.5 ± 11.6	98.0 ± 14.2	107.7 ± 12.1	1,2v3	9.8 (< 0.001)	0.16
%BF	24.8 ± 5.2	25.0 ± 4.1	30.9 ± 6.2	1,2v3	11.3 (< 0.001)	0.18
BFM [kg]	14.4 ± 4.7	15.4 ± 3.2	25.7 ± 7.5	1,2v3	40.1 (< 0.001)	0.43
LBM [kg]	42.4 ± 4.4	45.8 ± 4.6	56.4 ± 7.5	1v2v3	50.3 (< 0.001)	0.49
TBW [kg]	30.5 ± 3.2	33.0 ± 3.3	56.4 ± 7.5	1v2v3	50.5 (< 0.001)	0.49
SLM [kg]	39.1 ± 4.0	41.9 ± 4.8	51.7 ± 6.9	1v2v3	44.9 (< 0.001)	0.46
ICW	18.5 ± 2.0	20.0 ± 1.9	24.4 ± 3.2	1v2v3	50.0 (< 0.001)	0.49
ECW	12.0 ± 1.3	13.1 ± 1.5	16.1 ± 2.2	1v2v3	47.1 (< 0.001)	0.47

Notes: significant differences between groups (1 – endurance athletes; 2 – speed-power athletes, 3 – throwing athletes)

*BMD* Bone mineral density, *BMC* Bone mass, dis- distal part of forearm, *prox* proximal part of forearm, *BF* Body fat, *BFM* Body fat mass, *LBM* Lean body mass, *TBW* Total body water, *SLM* Soft lean mass, *ICW* Intercellular water, *ECW* Extracellular water

Backward stepwise logistic regression for the dependent variable BMD, with normal values depending on the sport is presented in the Tables 4 and 5. In men, the most important predictors of BMD (odds ratio - OR) in the distal segment were LBM (OR = 32.578; *p* = 0.023), ECW (OR = 0.003; *p* = 0.016), and ICW (OR = 0.011; *p* = 0.031). Furthermore, the analysis showed that in the proximal part, the most important predictors for normal BMD were SLM (OR = 5.008; *p* = 0.020) and ICW (0.354; *p* = 0.008). In women, the most important predictors of normal bone mineralization were ICW (OR = 10.174; *p* = 0.003) and LBM (OR = 0.470; p = 0.020) in the distal part and ICW (OR = 5.254; *p* = 0.038) in the proximal part.

**Table 4:** Multiple backward stepwise logistic regression in male masters athletes

PREDICTOR	ODDS RATIO	95% CI Upper	95% CI Lower	p	Chi^**2**^ Walda	R^**2**^ Nagelkerke
**NORM BMD distal**						
%BF	0,468	0,179	1223	0,121	2399	0,341
BFM	1850	0,647	5287	0,251	1319	
LBM	32,578	1629	651,604	0,023	5195	
ICW	0,011	0,000	0,672	0,031	4627	
ECW	0,003	0,000	0,347	0,016	5793	
BMI	1408	0,915	2167	0,119	2428	
**NORM BMD proximal**						
TBW	0,250	0,046	1372	0,111	2546	0,177
SLM	5008	1289	19,453	0,020	5415	
ICW	0,354	0,164	0,761	0,008	7069	

Notes: *BMD* Bone mineral density, *BF* Body fat, *BFM* Body fat mass, *LBM* Lean body mass, *ICW* Intercellular water, *ECW* Extracellular water, *BMI* Body mass index, *TBW* Total body water, *SLM* soft lean mass

**Table 5:** Multiple backward stepwise logistic regression in female masters athletes

PREDICTOR	ODDS RATIO	95% CI Upper	95% CI Lower	p	Chi^**2**^ Walda	R^**2**^ Nagelkerke
**NORM BMD distal**						
ICW	10,174	2223	46,565	0,003	8936	0,397
BFM	0,734	0,532	1012	0,059	3566	
LBM	0,470	0,249	0,888	0,020	5418	
BMI	1515	0,883	2601	0,132	2274	
Speed-power athletes	0,603	0,214	1699	0,166	1915	
Throws athletes	2204	0,222	21,879	0,349	0,876	
**NORM BMD proximal**						
ICW	5254	1099	25,112	0,038	4320	0,389
LBM	0,590	0,307	1134	0,114	2502	
Speed-power athletes	1859	0,389	8878	0,099	2729	
Endurance athletes	0,585	0,102	3365	0,186	1749	

Notes: *BMD* Bone mineral density, *ICW* Intercellular water, *BFM* Body fat mass, *LBM* Lean body mass, *BMI* Body mass index

## Discussion

This study used body composition and BMD data from different types of track and field athletes during the World Masters Athletics Championship to assess the condition of bone tissue of athletes aged 40 years and over. The results suggest that bone mineral density (BMD) and bone mass (BMC) in the forearm bone in masters athletes were strongly determined by the level of lean body components, intracellular water, extracellular water, and the type of training associated with the type of track and field event. Regardless of gender, athletes from throwing sports had the highest body mass and BMD of the forearm bones.

Masters athletics is becoming more and more popular because it gives the opportunity to actively participate in sports activities, compete with peers, and function by following the concept of active aging [20]. Research on physical activity and health-enhancing training of the population of older adults has been conducted mainly in terms of the effect on health and prevention of age-related chronic condition such as sarcopenia and osteoporosis [20, 21]. Athletes should pay much attention to their bone health both in terms of long-term (e.g. osteopenia and osteoporosis) and short-term bone injury risks [22]. The present data suggest that lifetime competitive activity has a positive effect on BMD and BMC in masters athletes.

The prevalence of osteoporosis in older adults has been widely demonstrated by numerous cross-sectional and longitudinal studies [23–25]. In the present study, no osteoporosis was observed in men in throwing events, and the lowest number of cases of osteoporosis in femoral neck and lumbar spine was observed among women in throwing sports (10.5%). This is more than 5% less than in Canadian studies of normal aging individuals, where the prevalence of osteoporosis (BMD T score ≤ − 2.5) in the femoral neck and lumbar spine in subjects aged 50 years and over was 6.6% in men and 15.8% in women [23]. However, the difficulty in comparing studies is caused by different skeleton locations in which BMD was evaluated, different ethnic groups, and differences in the methods of data presentation.

Apart from physical activity levels, the condition of bone tissue is determined by several other factors, including somatic variables and body composition. The current study showed significant differences in selected somatic features and body composition between masters athletes from three different types of track and field events. Compared to EA and SPA participants, male athletes of throwing sports were characterized by higher values of body height, body mass, wrist width, and forearm length. This is consistent with the previous research, confirming that strength and power training has a positive effect on bone status [12]. In addition, in male TA athletes, significantly higher (28–59%) values of individual body components were observed in relation to EA and SPA: extracellular water (ECW), total body water (TBW), lean body mass (LBM), soft lean mass (SLM), intercellular water (ICW) and body fat mass (BFM). In women, the analysis of the basic anthropometric characteristics of TA compared to EA and SPA athletes revealed higher body mass and wrist width. With regard to body components in the female TA group, higher (*p* < 0.001) values of TBW, LBM, ICW, ECW, SLM, BFM and percentage body fat (%BF) were observed compared to EA and SPA. Furthermore, in the female group, athletes from the SPA group were characterized by significantly higher values of LBM, TBW, ICW, ECW and SLM compared to EA.

Differentiation of the somatic structure and body composition of female and male athletes of different track and field events in the masters category has been mainly emphasized in terms of health and prevention of sarcopenia, osteoporosis in comparison to the health status of physically inactive peers [20]. Intensive exercise training in middle and old age helps maintain low body fat content, and this reduces the risk of heart disease and overweight and obesity-related diseases [14]. The present study showed that power events that require strength were associated with higher body mass and muscle mass which have been shown to reduce the risk of sarcopenia in older individual. This was confirmed by a previous study of male and female masters athletes with a mean age of 55.7 years who participated in the 2014 Pan Pacific Masters Games, where no case of sarcopenia was recorded [26].

Body composition analyses of masters athletes are considered ideal for gerontological and sarcopenic tests [27]. Given their level of sports and physical activity, masters athletes are assumed to be characterized by better health than their peers of the same age but less active [27]. The present study, the throwers had the highest body mass and lean body composition components, including muscle tissue, and this was significantly related to the highest BMC and BMD. These findings suggest that in late adulthood, physical training with elements of strength training and throwing with external load characteristic of weight, disc, hammer and ball has a significant effect on bone health and reduces the risk of osteopenia and osteoporosis in aging individuals [28, 29]. It is also possible that the masters athletes included in the current study also trained in their youth. This may have had an effect on peak bone mass and the preservation of bone status for later years of life.

A number of previous cross-sectional studies on adults in different age have been conducted on the evaluation of BMD in relation to physical activity, but their results are inconclusive. Several studies have found a positive relationship between BMD and regular lifelong physical activity [30, 31]. However, the BMD change per standard deviation of physical activity was only < 1% or accounted for a small part of the variance. In subsequent studies, physical activity was related to the BMD norm but depended on other factors [32]. It is difficult to compare studies because of the differences in their duration, accuracy, and methods used to measure the level of physical activity, and type of physical exercise.

In our research, for a better understanding of the factors affecting BMC and BMD of masters athletes, a logistic regression analysis was conducted for the dependent variable BMD. It was demonstrated that in male athletes, the most important predictors of the BMD norm were tissue components of the body, especially LBM (OR = 32.578), hydration components ECW and ICW in the distal part and SLM (OR = 5.008) and ICW in the proximal part. In female athletes, the most important predictors of BMD were ICW (OR = 10.174) and LBM (OR = 0.470) in the distal part and ICW (OR = 5.254) in the proximal segment.

The risk factors for low BMD after age of 50 years and older often include analyses based only on body mass and BMI without a thorough analysis of specific body composition components. Numerous studies from China, the USA, and Europe have shown a positive significant dependence of BMD on body mass and BMI with BMD between 3 to 7% higher in the hip and lumbar region per 10 kg of body mass increase [30–33].

The present study of masters athletes, BMD and BMC were significantly related to lean body composition components and, interestingly, by intracellular and extracellular water. Both adipose tissue and lean body mass can affect bone mass [21] with their relative effect modulated by their absolute amount and ratio to total body mass. The meta-analysis by Ho-Pham and his colleagues [34] demonstrated that tissue components have a significant impact on bone condition but it depends on the skeleton measurement location. Moreover, the positive character of the lumbar spine BMD correlation with lean body mass was demonstrated after taking into account age and body mass [24, 25]. The effect of lean body mass on higher BMD and BMC at all skeletal locations in the current study may be explained by the associated bone loading and its effect on the biomechanical relationships between body size and bone mass [35]. Relatively high mechanical load reduces bone resorption and stimulates bone formation, increases bone strength and mineral content, and delays the occurrence of osteoporosis [35]. This explain the results obtained for both BMD and BMC in the present study. These findings may an agreement with Tanaka et al. [36] who stated that masters athletes have greater functional capacity at any age than their sedentary peers. However, it should be noticed that only healthy aging adults with healthy body composition and good physical function typically to participate in competitive masters sports [26].

Water is an important constituent of the body so changes in the total body water (TBW) will impact on body composition. The differentiation of TBW into intracellular water (ICW) and extracellular water (ECW) compartments is useful to describe fluid shifts and fluid balance and to explore variations in levels of hydration [37].

Water makes up about a 1/4 of a bone’s mass [38]. Bone is also a fluidimbibed material in which the distribution of water affects the mechanical properties of bone. Collagen and calcium account for the rest of the mass. When bones contain enough water, nutrients are able to target bone tissue more effectively. Studies also shown that water is a key component in the function of the cortical bone, which is the hard external layer of the bone. Although the effects of dehydration on the mechanical behavior of cortical bone are known [39], the underlying mechanisms for such effects are not clear. From an energy perspective, the research focus to water–mineral interaction and the water–collagen interaction. Therefore, scientists speculate that loss of water in the collagen phase decreases the toughness of bone, whereas loss of water associated with the mineral phase decreases both bone strength and toughness [39]. In our study it was demonstrated that in the male group, the most important predictors of the BMD norm were tissue components of the body, among them also hydration components ECW and ICW in the distal part and ICW in the proximal part. In women also in the most important predictors were ICW (OR = 10.174) in the distal part and ICW (OR = 5.254) in the proximal segment.

Previous bone tissue studies have emphasized positive correlations of BMD with body mass, with calcium 50 to 70% of body mass in a healthy adult being water, which facilitates the transport of nutrients to body cells. Therefore, the dependence of BMD and BMC on the appropriate levels of TBW, ECW, and ICW may be explained in the role of transporting body fluids for appropriate trophic activity and nourishing bone tissue. Furthermore, in the extracellular fluid, albumin is the most abundant protein and accounts for calcium 70% of the colloid osmotic pressure in plasma and thus plays an important role in regulating the distribution of the fluid in the human body, including bone tissue [40, 41]. However, the nature and relationships between the level of body hydration and bone mineral status further research.

The type of activity appears to strongly improve on BMD. However, most studies have been carried out in groups of adolescent. This effect was also confirmed on aging active people remains unclear. It can be assumed that weight-bearing activities are an important determinant of bone density. High-impact training including sprinting, throwing and jumping also seems to be associated with the modification of the bone structure by having great osteogenic potential [42, 43]. In Masters Athletes, Piasecki et al. [13, 14] stated that especially sprinting is associated with greater hip, spine and tibial BMD. What is more this effect was not confirmed to endurance running. It strongly suggest that aerobic activity should also be supplemented by dynamic sprint or jumping activities. Furthermore, activity during growth and young adulthood periods results in improvements in bone density in middle-aged and older adults [44]. The present findings are in agreement with previous research. A study of women in the post-menopausal involutional age showed that the highest BMD and BMC values were found in women who were physically active throughout their lives [16]. In the present study, the participants were physically active and involved in sports training in the first and second decades of life. This observation suggest that prior sports training influences peak bone mass and in later decades.

One limitation of the full interpretation of the results of the study is the relatively small number of athletes studied after taking into account gender and the type of track and field event. The findings of the study suggest the need for bone scans in other skeletal locations of older athletes.

## Conclusions

The prevalence of low T-scores in the form of osteopenia and osteoporosis especially among women (EA,SPA,TA) in both measurement sections, and in men (EA and SPA) especially in the proximal section indicate the presence of developing osteoporosis risk which might lead to fractures in more than half of the masters athletes. The exception is the TA group of men. The representatives of strength events had the most advantageous BMD levels. Therefore, strength based exercises are suggested to slow the process of osteopenia and osteoporosis. The condition of bone tissue evaluated by bone mass (BMC) and bone mineral density (BMD) of the forearm in masters athletes was strongly determined by the level of lean body components and the type of sports training associated with the different track and field events. The dependence of BMD on tissue components ICW and ECW in aging athletes is an important finding. The most important predictors of the BMD norm were also hydration components ECW and ICW. Intracellular and extracellular water levels increased the odds ratio of normal bone mineralization by several times. However, this relationship requires ​more research as to delineate the nature and mechanisms of these interactions.

## Data Availability

The datasets generated and/or analysed during the current study are not publicly available due to institutional restrictions but are available from the corresponding author on reasonable request.

## Abbreviations

BMC: Bone mass
BMD: Bone mineral density
DIS: Distal part of forearm
EA: Endurance athletes
ECW: Extracellular water
ICW: Intracellular water
LBM: Lean body mass
BFM: Mass of body fat
%BF: Percentage body fat
PROX: Proximal part of forearm
SLM: Soft lean mass
SPA: Speed-power athletes
TA: Throwing athletes
TBW: Total body water
