# Correction to: Bone mineral density in elite masters athletes: the effect of body composition and long-term exercise

**DOI:** 10.1186/s11556-021-00267-9

**Published:** 2021-07-03

**Authors:** Anna Kopiczko, Jakub Grzegorz Adamczyk, Karol Gryko, Marek Popowczak

**Affiliations:** 1 Department of Human Biology, Józef Piłsudski University of Physical Education in Warsaw, Marymoncka 34, 00-968 Warsaw, Poland; 2 Department of Theory of Sport, Józef Piłsudski University of Physical Education in Warsaw, Marymoncka 34, 00-968 Warsaw, Poland; 3 Department of Sport Games, Józef Piłsudski University of Physical Education in Warsaw, Marymoncka 34, 00-968 Warsaw, Poland; 4 Department of Team Sport Games, University School of Physical Education in Wrocław, Al. Ignacego Jana Paderewskiego 35, 51-612 Wrocław, Poland

**Correction to: Eur Rev Aging Phys Act 18, 7 (2021)**

**https://doi.org/10.1186/s11556-021-00262-0**

Following the publication of the original article [1] the authors noticed that Tables 4 and 5 in some place dots are missing; therefore, some values are not true.

The original article [1] has been updated.

The correct Tables 4 and 5 are shown below.

**Table 4:** Multiple backward stepwise logistic regression in male masters athletes

PREDICTOR	ODDS RATIO	95% CI Upper	95% CI Lower	p	Chi^2^ Walda	R^2^ Nagelkerke
**NORM BMD dis.**						
PBF	0.468	0.179	1.223	0.121	2.399	0.341
MBF	1.850	0.647	5.287	0.251	1.319	
LBM	32.578	1.629	651.604	0.023	5.195	
ICW	0.011	0.000	0.672	0.031	4.627	
ECW	0.003	0.000	0.347	0.016	5.793	
BMI	1.408	0.915	2.167	0.119	2.428	
**NORM BMD prox.**						
TBW	0.250	0.046	1.372	0.111	2.546	0.177
SLM	5.008	1.289	19.453	0.020	5.415	
ICW	0.354	0.164	0.761	0.008	7.069	

**Table 5:** Multiple backward stepwise logistic regression in female masters athletes

PREDICTOR	ODDS RATIO	95% CI Upper	95% CI Lower	p	Chi^2^ Walda	R^2^ Nagelkerke
**NORM BMD dis.**						
ICW	10.174	2.223	46.565	0.003	8.936	0.397
MBF	0.734	0.532	1.012	0.059	3.566	
LBM	0.470	0.249	0.888	0.020	5.418	
BMI	1.515	0.883	2.601	0.132	2.274	
Speed-power athletes	0.603	0.214	1.699	0.166	1.915	
Throws athletes	2.204	0.222	21.879	0.349	0.876	
**NORM BMD prox.**						
ICW	5.254	1.099	25.112	0.038	4.320	0.389
LBM	0.590	0.307	1.134	0.114	2.502	
Speed-power athletes	1.859	0.389	8.878	0.099	2.729	
Endurance athletes	0.585	0.102	3.365	0.186	1.749	
